# Surfacing the deep data of taxonomy

**DOI:** 10.3897/zookeys.550.9293

**Published:** 2016-01-07

**Authors:** Roderic D. M. Page

**Affiliations:** 1 Institute of Biodiversity, Animal Health, and Comparative Medicine, College of Medical, Veterinary, and Life Sciences, University of Glasgow, Glasgow G12 8QQ, UK

**Keywords:** Biodiversity informatics, identifiers, DOI, literature, taxonomy, dark taxa, data cleaning, data integration

## Abstract

Taxonomic databases are perpetuating approaches to citing literature that may have been appropriate before the Internet, often being little more than digitised 5 × 3 index cards. Typically the original taxonomic literature is either not cited, or is represented in the form of a (typically abbreviated) text string. Hence much of the “deep data” of taxonomy, such as the original descriptions, revisions, and nomenclatural actions are largely hidden from all but the most resourceful users. At the same time there are burgeoning efforts to digitise the scientific literature, and much of this newly available content has been assigned globally unique identifiers such as Digital Object Identifiers (DOIs), which are also the identifier of choice for most modern publications. This represents an opportunity for taxonomic databases to engage with digitisation efforts. Mapping the taxonomic literature on to globally unique identifiers can be time consuming, but need be done only once. Furthermore, if we reuse existing identifiers, rather than mint our own, we can start to build the links between the diverse data that are needed to support the kinds of inference which biodiversity informatics aspires to support. Until this practice becomes widespread, the taxonomic literature will remain balkanized, and much of the knowledge that it contains will linger in obscurity.

Digital Object Identifiers

## Introduction

Bergman (2001) coined the term “deep web” to refer to the part of the web that is largely hidden from search engine crawlers, such as dynamically generated web pages. A major challenge facing web crawlers is how to “surface” that deep web so that it becomes accessible to search engines. By analogy, while much of the scientific literature is readily discoverable, taxonomic literature remains largely obscure.

As an example of the consequences of this obscurity, consider the fate of the name *Leviathan* as used for a recently discovered fossil whale described in *Nature* (Lambert et al. 2010a). Unbeknownst to the authors, the name *Leviathan* was previously used for an extinct mammoth (Koch 1841). Once the homonymy was uncovered, an erratum was published in the same journal (Lambert et al. 2010b). The erratum cites the original publication of *Leviathan* (Koch 1841), but if the reader visits the corresponding page on the journal *Nature*’s website there is no link to the digitised version of this publication, despite it being available in the Biodiversity Heritage Library (http://biodiversitylibrary.org/). The lack of a link is symptomatic of the poor state of digital access to taxonomic literature. Reading the list of literature cited in a modern taxonomic paper online, it is striking that while citations of papers on phylogenetics, ecology, or methodology typically include links directly to that article (for example, using Digital Object Identifiers, DOIs), the citations to taxonomic literature are mostly devoid of such links. In an age when most readers will expect any publications worth reading to be online, the absence of links to the primary taxonomic literature consigns it to a ghetto where only the most determined and well-resourced readers will dare to venture. For many readers the lack of a link means a dead-end in their search for information.

Unless we want the taxonomic literature to linger in obscurity we need to make it easily findable and accessible. An obvious starting point would be if taxonomic databases linked to the digitised taxonomic literature. However, most taxonomic databases are little more than online collections of 5 × 3 index cards, a technology Linnaeus himself pioneered (Müller-Wille and Charmantier 2011). Often databases simply present the user with lists of names, divorced from the associated taxonomic literature (such as the original publication of the name). When literature is cited, it is typically as a text string, lacking either an identifier or a link that the researcher can use to locate the publication. This is not to deny the value of the heroic efforts of indexers such as Charles Davies Sherborn (Cleevely 2009), but it is striking how persistent conventions from the print world remain, despite the Internet removing many of the physical constraints of that medium. For example, the recent publication of the Rotifer List of Available Names (LAN) (Segers et al. 2012) comprises a list of references in abbreviated form (“microcitation”) instead of the full publication details, and the list lacks any bibliographic identifiers. Part of the goal of publishing such a List of Available Names is to enable others to scrutinise it, yet the authors of the list provide virtually no assistance to the reader in locating the corresponding literature.

If we accept that the key documents of taxonomy are the publications that contain the names, descriptions, nomenclatural changes, and taxonomic revisions, then a major challenge is to “surface” these documents so that readers can discover them. This means changing practices that have served the community well in the pre-digital era, but which are now hindering its progress. One of the key changes will be the adoption of globally unique identifiers for the taxonomic literature.

## Globally unique identifiers

The taxonomic community’s experience with globally unique identifiers has been mixed. Several factors have contributed to this. The first is the saga of Life Science Identifiers (LSIDs) (Martin et al. 2005) which seemed a promising technology for identifying data in biology, but in the end the biodiversity community were the only major adopters. This was compounded by the lack of reusing existing identifiers. Every project employing LSIDs created their own identifiers for their data, and rarely, if ever, used LSIDs from other projects. For example, both the Index of Organism Names (ION, http://www.organismnames.com/) and ZooBank (http://zoobank.org/) have records for the genus name *Tyrannobdella*, each with their own LSID (urn:lsid:organismnames.com:name:4439403 and urn:lsid:zoobank.org:act:43D55B49-C888-4D6B-AF6F-61238EC1339B, respectively). Neither database acknowledges the existence of the other by using the other’s identifier. Furthermore, neither ION nor ZooBank use the most obvious identifier for the PLoS One paper that published *Tyrannobdella* (Phillips et al. 2010), namely the DOI: 10.1371/journal.pone.0010057. ION represents the reference as a text string:

“Tyrannobdella rex n. gen. n. sp. and the evolutionary origins of mucosal leech infestations. PLoS ONE, 5(4) 2010: e1057, 1–8.”

ZooBank mints its own identifier for the PLoS One paper: urn:lsid:zoobank.org:pub:8D431ED1-B837-4781-A591-D3886285283A (since this was written ZooBank has added the DOI for this article). Ironically, the only thing that links these two records together is the taxonomic name “*Tyrannobdella*”.

A consequence of the failure to reuse existing identifiers is that the biodiversity informatics community has created a large amount of data identified by a technology few people understand (LSIDs, which by default wouldn’t work in a web browser) and with very few cross-links. This lack of links means each database is effectively another silo, and hence many of the expected benefits of serving biodiversity data in RDF (Page 2006) have not materialised.

This experience may encourage a healthy scepticism about the utility of identifiers, but I would argue that this is because we’ve overlooked the importance of their reuse. If different databases insist on minting their own identifiers and not using (or linking to) existing identifiers, then our data will remain in silos. Reusing identifiers will help establish links between databases, and it is these links that will be the basis of many of the hoped-for inferences we can make in biodiversity informatics (Page 2008).

### Bibliographic identifiers

Taxonomic databases often contain names devoid of references to the literature. Names by themselves are of little value; it is the literature, specimens, and data derived from those specimens that are the primary data of taxonomy. Yet much of this information remains hard to obtain (even discovering that it exists can be challenging). Many citations to the taxonomic literature are obscure unless you are familiar with the conventions. For example, if you are searching for the original publication of the name *Tachyglossus* Illiger, 1811 (a genus of spiny anteaters) then *Nomenclator Zoologicus* (Neave 1939; Remsen et al. 2006) gives this as “Prod., 114.” I suspect that most readers will find this less than helpful. The citation refers to page 114 of “Caroli Illigeri D. Acad. Reg. Scient. Berolinens. et Bavaricae Sod. Museo Zoologico Berolin. praefecti professoris extraord. Prodromus systematis mammalium et avium : additis terminis zoographicis utriusque classis, eorumque versione germanica.” Given the length of the title of Illiger’s work, one can see the desirability of abbreviating it for a printed list such as *Nomenclator Zoologicus*. But there are many ways to abbreviate a citation, which can result in a plethora of ways the same publication is cited in different databases (sometimes even within the same database).

One approach to tackling the plethora of ambiguous, if not downright obscure, citations is to use globally unique identifiers to refer to the publications. In the case of the “Prodromus systematis mammalium et avium” (Illiger 1811), this publication has recently acquired a DOI (10.5962/bhl.title.42403) assigned by the Biodiversity Heritage Library. DOIs are widely used in the publishing industry to identify articles (such as this the one you are currently reading), and are increasingly being used as identifiers for other digital objects, such as data sets (e.g., the DataCite project http://datacite.org/). By providing unique, stable identifiers for articles, the publishing industry has simplified the task converting lists of literature cited into clickable links. DOIs have been in use to identify the scientific literature for over a decade, but taxonomic databases have been slow to adopt these identifiers.

### The utility of identifiers

Using existing bibliographic identifiers has several immediate advantages. It all but eliminates ambiguity in citations. Given that the same citation can be represented multiple ways (consider the bewildering and completely unnecessary proliferation of citation styles for different journals), matching citations using their representation as strings of characters is fraught with problems. Citation strings can also “mutate” over time (Specht 2010) and these mutations can propagate by “copy and paste” citation (Simkin and Roychowdhury 2011). Consistent use of globally unique identifiers mitigates this problem.

Identifiers provide additional value if they come with supporting services. For example, DOIs can be resolved to both human- and machine-readable content, which enables tools to be built that can consume DOIs and automatically populate databases with bibliographic information (most bibliographic management software makes use of these services). There are also services that take a bibliographic citation and find the corresponding DOI; publishers utilise these to add links to the list of literature cited in an article.

But the real value from identifiers becomes apparent when they are shared, that is, when different databases use the same identifiers for the same entities, instead of minting their own. Reusing identifiers can enable unexpected connections between databases. For example, the PubMed biomedical literature database has a record (PMID:948206) for the paper “Monograph on “*Lithoglyphopsis*” *aperta*, the snail host of Mekong River Schistosomiasis” (Davis et al. 1976). The PubMed record contains the abstract for the paper, but no link to where the user can obtain a copy of the paper. Actually, this reference is in a volume scanned by BHL, and has been extracted by BioStor (http://biostor.org/reference/102054). If PubMed was linked to BHL, users of PubMed could go straight to the content of the article. But this is just the start. The Davis et al. (1976) paper also mentions museum specimens in the collection of the Academy of Natural Sciences of Drexel University, Philadelphia. Metadata for these specimens has been aggregated by GBIF, and the BioStor page for this article displays those links. In an ideal world we should be able to go from PubMed to BioStor to GBIF. But in many ways the real power will come from traversing these links in the other direction. At present, a user of GBIF simply sees metadata for these specimens and a locality map. They are unaware that these specimens have been cited in a paper (Davis et al. 1976) which shows that the snails host the Mekong River schistosome. This connection would be trivial to make if the reciprocal link was made from GBIF to BioStor. Furthermore, the link from BioStor to PubMed would give us access to Medical Subject Headings (MeSH http://www.nlm.nih.gov/mesh/) for the paper. Hence we could imagine ultimately searching GBIF using queries from a controlled vocabulary of biomedical terms.

Making these connections requires not only that we have digital identifiers, but also that wherever possible we reuse existing identifiers. If we restrict ourselves to project-specific identifiers then we stymie attempts to create a network of connected data on biodiversity.

It is worth exploring ways we can reuse identifiers. One approach is to include links to existing identifiers wherever possible. For example, if a database includes an article that has a DOI, then that database should store the DOI as one of its fields. This is the easiest form of reuse, and doesn’t prevent the database minting its own identifiers. This approach makes sense if we are adding data that hasn’t yet been linked to existing identifiers, or if identifiers may only become available later (e.g., after a database entry has been created, a publisher subsequently digitises the print archive of a journal and issues DOIs for each article). A more powerful example of reuse is when a database incorporates existing identifiers into its own identifiers. The BBC is an excellent example of this: their music and nature sites reuse “slugs” from external resources, such as MusicBrainz and Wikipedia, respectively (Raimond et al. 2010). The “slug” is the part of the URL after the domain name (and any site-specific details). Hence, given that the URL for the Wikipedia page for the Komodo dragon (*Varanus komodoensis*) is http://en.wikipedia.org/wiki/Komodo_dragon, the BBC reuse the slug “Komodo_dragon” to create the URL http://www.bbc.co.uk/nature/life/Komodo_dragon. Similarly, instead of minting a completely new identifier for a journal, we can make use of the journal’s ISSN to create a URL (e.g., http://bionames.org/issn/1313-2989). Reusing identifiers in this manner makes it easier to find equivalent entries in different databases (Raimond et al. 2010).

### Identifiers and community

”This may not be much of a revelation to many, but is a notion that is sinking home more deeply for me of late. By “Community”, I don‘t necessarily mean the online community … I mean the taxonomic community.” David Shorthouse “The community is dead” http://ispiders.blogspot.co.uk/2009/06/community-is-dead.html

There are many reasons why communities may or may not form, but arguably a community that shares an interest in a given topic benefits from having a standard way to refer to the things they care about. The increasing adoption of standard bibliographic identifiers such as DOIs makes it easier to build social bookmarking tools around the scientific literature (such as CiteULike http://www.citeulike.org/ and Mendeley http://www.mendeley.com/) because it becomes easier to determine how many members of the network have bookmarked the same paper.

Taxonomic communities are likely to be small and taxon-focussed. But this does not mean that these are the only communities that taxonomists can engage with, or that people outside the taxonomic community will not share the interests of those working on a particular taxon. Using bibliographic identifiers we can discover networks of people interested in particular topics that may intersect with taxonomists (obvious examples are people interested in ecology, conservation and evolutionary biology). By making publications the unit of sharing, companies such as Mendeley have grasped perhaps better than most that the connection between researchers is often not a direct social link, but rather shared interest in the same publication (formalised by patterns of citation and co-citation). For this reason, I suspect that attempts to build communities around taxa (Harman et al. 2009) may be ultimately less successful than embedding the taxonomic literature in the growing social networks assembling around scientific publications.

### Identifiers and impact

The taxonomic community has long felt disadvantaged by the role of citation-based “impact factor” in assessing the importance of taxonomic research (Garfield 2001; Krell 2000; Werner 2006) especially as much of the taxonomic literature appears in relatively low impact journals. A common proposal is to include citations to the taxonomic authority for every name mentioned in a scientific paper (Wägele et al. 2011). Regardless of the merits of this idea, the difficulty of locating bibliographic details for much of the taxonomic literature, coupled with the lack of identifiers such as DOIs means such proposals will be hard to implement, and likely to merely populate the literature cited section of papers with even more bibliographic dead ends.

At the same time, the concern about impact may help motivate the use of identifiers such as DOIs. There is a growing “altmetrics” movement (http://altmetrics.org/manifesto/) that aims to provide metrics for the post-publication impact of a publication in terms of activity such as social bookmarking, and commentary on web sites (Yan and Gerstein 2011). Gathering these metrics is greatly facilitated by using standard bibliographic identifiers (otherwise, how do we know whether two commentators are discussing the same article or not?). If taxonomic literature is be part of this burgeoning conversation it needs to be able to be identified unambiguously.

## Making the taxonomic literature findable

The first step towards improving the current generation of taxonomic databases would be to associate the taxonomic literature with existing digital identifiers, such as DOIs. Admittedly, this will not always be straightforward. Although DOIs are the bibliographic identifier of choice, and CrossRef provides tools for locating an existing DOI for a reference, it is not always straightforward to find a DOI for a publication. Part of the difficulty in citing the older literature is that many of the conventions we take for granted in modern scientific articles are lacking. Modern articles have titles, and are published in journals that usually have an unambiguous name, volume number, and pagination. This triplet is usually unique, and makes it relatively easy to locate an article in a bibliographic database (Page 2009). However, these conventions need not apply to older publications. For example, (Bennett and Jarvis 2004) cite the following paper:

Ogilby W (1838) On a collection of Mammalia procured by Captain Alexander during his journey into the country of the Damaras. Proceedings of the Zoological Society of London 1838:5–15.

This journal has been digitised by both Wiley and BHL. Wiley makes pages 5-15 available as an article with the doi: 10.1111/j.1096-3642.1838.tb01402.x and attributes the authorship to Richard Owen, not W. Ogilby. On inspection we see that pages 5–15 comprise two articles, one by Ogilby and one by Owen. The first paragraph of page 5 contains the text:

“A selection of the Mammalia procured by Captain Alexander during his recent journey into the country of the Damaras, on the South West Coast of Africa, was exhibited, and Mr. Ogilby directed the attention of the Society to the new and rare species which it contained.”

Subsequent authors have transformed this sentence into the article title “On a collection of Mammalia procured by Captain Alexander during his journey into the country of the Damaras”. Note also that in this case, there is a mismatch between the granularity at which taxonomists cite the literature and the granularity at which Wiley has assigned the identifier (the DOI corresponds to two articles). Perhaps the most obvious example of this mismatch is exemplified by the BHL, which typically recognises units at the scale of journal volume, or individual pages, but not at article level (Page 2011a).

Discovering existing identifiers for the taxonomic literature will sometimes be difficult, for a multitude of reasons. For example, taxonomic databases often store an abbreviated (or even corrupted) version of the citation, the citation may be translated from its original language, or the journal may have been renamed and the new name applied retrospectively to older issues (Page 2011c). All of this makes creating the mapping tedious, but this mapping need only be done once.

### Kinds of identifiers

While DOIs are the best-known bibliographic identifier, there are several others that are relevant to the taxonomic literature (Page 2009). DOIs are themselves based on Handles (http://hdl.handle.net) an identifier widely used by digital repositories such as DSpace (http://www.dspace.org/). A number of journals, such as the *Bulletins* and *Novitates of the American Museum of Natural History* are available in DSpace repositories and consequently have Handles. Other major archives such as JSTOR (http://www.jstor.org/) and CiNii (http://ci.nii.ac.jp/) have their own unique identifiers (typically integer numbers that are part of a URL). Having a variety of identifiers complicates the task of finding existing identifiers for a particular publication. Whereas for some identifiers, such as DOIs and CiNii NAIDs, (National Institute of Informatics Article IDs) there are OpenURL resolvers for this task, for other identifiers there may be no obvious way to find the identifier other than by using a search engine.

Identifiers also exist for aggregations of publications, such as journals. The practice of abbreviating journal titles has led to a plethora of ways to refer to the same journal. For example, the BioStor database (Page 2011a) has the following entries for the *Bulletin of Zoological Nomenclature*:

Bulletin of Zoological Nomenclature

The Bulletin of Zoological Nomenclature

Bull. Zool. Nom.

Bull.zool. Nom.

Bull. Zool. Nom

Bull, Zool. Nom.

Bull Zool. Nom.

Bull. Zool.nom.

Bull. Zool Nom.

Bull., Zool. Nom.

Bull. Zool. . Nom.

Bulletin Zoological Nomenclature

Bull Zoological Nomenclature

Bull Zool Nomen

Bull. Zool. Nomencl

Bull Zool Nom.

Bulletin of Zoological Nomeclature

Bulletin Zool. Nom.

Bull. Zool. Nomencl.

This practice of abbreviating journal names (motivated by the desire to conserve space on the printed page) complicates efforts to match citations to identifiers. One approach to tackling this problem is to map abbreviations to journal-level globally unique identifiers, such as International Standard Serial Numbers (ISSNs) (for the *Bulletin of Zoological Nomenclature* the ISSN is 0007-5167). In addition to reducing ambiguity, there are web services that take ISSNs and return the history of name changes for a journal, which in turn can help clarify the (often complicated) history of long-lived journals.

### How much taxonomic literature has been digitised?

To assess the extent of taxonomic digitisation I harvested the metadata associated with the LSID for each record in the ION database. This database records names published under the International Code of Zoological Nomenclature. Over 4 million records have been harvested and imported into BioNames (http://bionames.org) (Page 2013), over a million of which have an associated bibliographic citation. In order to locate identifiers for these citations I attempted to parse each one into its constituent components (e.g., title, journal, volume, pagination) and used OpenURL resolvers to find the corresponding record in databases such as CrossRef and BioStor. To complement this approach I have harvested metadata for some 300,000 journal articles and stored these in Mendeley, then used approximate string matching to compare these to records in ION. This work is on-going, current results can be seen at http://bionames.org/dashboard. To date BioNames has over 60,000 articles with DOIs that publish new names, and if we consider all potential bibliographic identifiers (DOIs, Handles, PubMed, URLs, PDFs) then approximately 20% of all ICZN names are linked to publications that have a digital presence.

### Access to the literature

Of course, having the literature digitised is not the same as having ready access to it. Numerous parties are undertaking digitisation efforts, and the results are being made available under a wide range of conditions. Some output is available under explicitly open access licenses (MacCallum 2007), such as content from BHL and the journals published by Pensoft and the Public Library of Science. Some publishers, notably Taylor and Francis, and Wiley are digitising back catalogues of journals and making them available to subscribers. Archives such as JSTOR and CiNii have a mixture of free and subscription-based content. Many smaller journals, often published by scientific societies are providing their contently for free online, if not explicitly under an open license. Note that it is something of a misconception that the bulk of BHL’s content is pre-1923. In fact, for several key taxonomic journals its coverage extends into the 21^st^ century, in places overlapping with content made available by the original publishers.

## Discussion

As a final motivation to surface deep taxonomic data, consider the rise of “dark taxa” in genomics databases (Page 2011b). A growing percentage of “taxa” in GenBank lack a formal scientific name; in 2010 dark taxa comprised over 80% of invertebrate taxa added that year (Parr et al. 2011). Many of the most recent dark taxa are a product of DNA barcoding projects, and at the time of writing these sequences have been “suppressed” by GenBank, that is, they are still in the database but do not feature in search results. But there is still a background trend towards increasing numbers of unidentified sequences in GenBank. A significant challenge will be determining whether these dark taxa represent newly discovered taxa, or come from known taxa but have not been identified as such (Hibbett and Glotzer 2011; Nagy et al. 2011).

It is clear that some dark taxa do, in fact, have names. For example, consider the frog “Gephyromantis aff. blanci MV-2005” (NCBI tax_id 321743), which has a single sequence AY848308 associated with it. This sequence was published as part of a DNA barcoding study (Vences et al. 2005). If we enter the accession number AY848308 into Google we find two documents, one the supplementary table for (Vences et al. 2005), the other the a subsequent paper by (Vences and Riva 2007) that describes the frog with this sequence as a new species, *Gephyromantis runewsweeki*. This is a relatively straightforward example, and the taxonomic description is freely available online. But it still required significant time to track down the species description for this one example.

A key question facing attempts to find names for dark taxa is whether the methods available can be scaled to handle the magnitude of the problem. One could argue that newer technologies such as DNA barcoding make classical taxonomy less relevant, and perhaps the effort in digitising older literature and exposing the taxonomic names it contains is misplaced. A counter argument would be that the taxonomic literature potentially contains a wealth of information on ecology, morphology and behaviour, often for taxa in areas that have been subsequently altered by human activity. Furthermore, as technologies such as barcoding uncover previously overlooked variation, older taxonomic names previously sunk in synonymy may yet become relevant. For example, several taxa have been synonymised with the silvery mole-rat *Heliophobius argenteocinereus* Peters, 1846 (Peters 1846) but DNA sequence data has revealed several clades within that species (Faulkes et al. 2011). Consequently, rather than coin new names for these clades we can rescue older names from synonymy. Hence DNA barcoding may give a new lease of life to old names.

Names may have a special place in the hearts of taxonomists (Patterson et al. 2010) but the pace of biodiversity discovery is outstripping our ability to put names on taxa, as evidenced by the rise of dark taxa in GenBank. There are increasing calls to adopt less formal taxonomic naming schemes (Schindel and Miller 2010), or to focus on describing biodiversity without necessarily naming it (Deans et al. 2012; Maddison et al. 2011). Underpinning much of this call to “ramp up” the rate of biodiversity description will be identifiers, assigned to the entities that taxonomy deals with, including specimens, genotypes, phenotypes, publications, and, yes, taxonomic names. As I have argued previously (Page 2008), in many ways taxonomists have been doing this already but without using web-friendly identifiers. Examples include lists of collection acronyms (Leviton et al. 1985) and author names. The issue now is how do we scale these activities to accommodate the deluge of data we are accumulating as we digitise life and our efforts to document it?
